# Recurrence of an undifferentiated pleomorphic pulmonary artery sarcoma 8 years after initial presentation: a case report

**DOI:** 10.3389/fcvm.2024.1378333

**Published:** 2024-06-25

**Authors:** Baudouin Bourlond, Niccolo Maurizi, Panagiotis Antiochos, Ioannis Skalidis, Katarina Auf Der Springe, Claire Royer, Pierre Monney, Olivier Muller, Mathias Kirsch

**Affiliations:** ^1^ Department of Cardiology, Lausanne University Hospital (CHUV), Lausanne, Switzerland; ^2^ Department of Nuclear Medicine and Molecular Imaging, Lausanne University Hospital and University of Lausanne, Lausanne, Switzerland; ^3^ Department of Anatomopathology, Lausanne University Hospital (CHUV), Lausanne, Switzerland

**Keywords:** cardiac sarcoma, cardiac MRI, pulmonary artery sarcoma, sarcoma, cardio-oncology, case report

## Abstract

**Background:**

Primary cardiac tumors remain exceptionally rare, characterized by a poor prognosis. Among them, sarcomas originating in the pulmonary arteries constitute the most infrequent subgroup within primary cardiac sarcomas.

**Case summary:**

This report presents the case of a 76-year-old female experiencing a recurrence of an undifferentiated pleomorphic intracardiac pulmonary artery sarcoma located in the right ventricular outflow tract, manifesting 8 years after initial remission. Successful outcomes were attained through a combination of surgical resection, state-of-the-art radiotherapy, and chemotherapy. This comprehensive approach proved essential for optimizing both survival and quality of life.

**Discussion:**

The unexpectedly prolonged recurrence-free survival observed in this case underscores the effectiveness of the comprehensive multimodal treatment approach outlined in the existing literature. This highlights the pivotal role of a multidisciplinary strategy in addressing primary cardiac sarcomas, particularly those arising in the pulmonary arteries.

## Introduction

Primary cardiac tumors are exceptionally rare and associated with a dismal prognosis. Among these, sarcomas originating in the pulmonary arteries constitute the rarest subset of primary cardiac sarcomas. The symptoms are nonspecific and can closely resemble those of various other cardiac diseases. Diagnosing these tumors remains a formidable challenge and is frequently prone to misidentification. Multimodality imaging plays a pivotal role in the diagnostic process, although the definitive diagnosis is ultimately established through anatomopathological examination.

## Case report

### Patient presentation

We present a case involving a 76-year-old female who experienced a recurrence of undifferentiated pleomorphic pulmonary artery sarcoma, 8 years after achieving remission.

**Figure 1:**
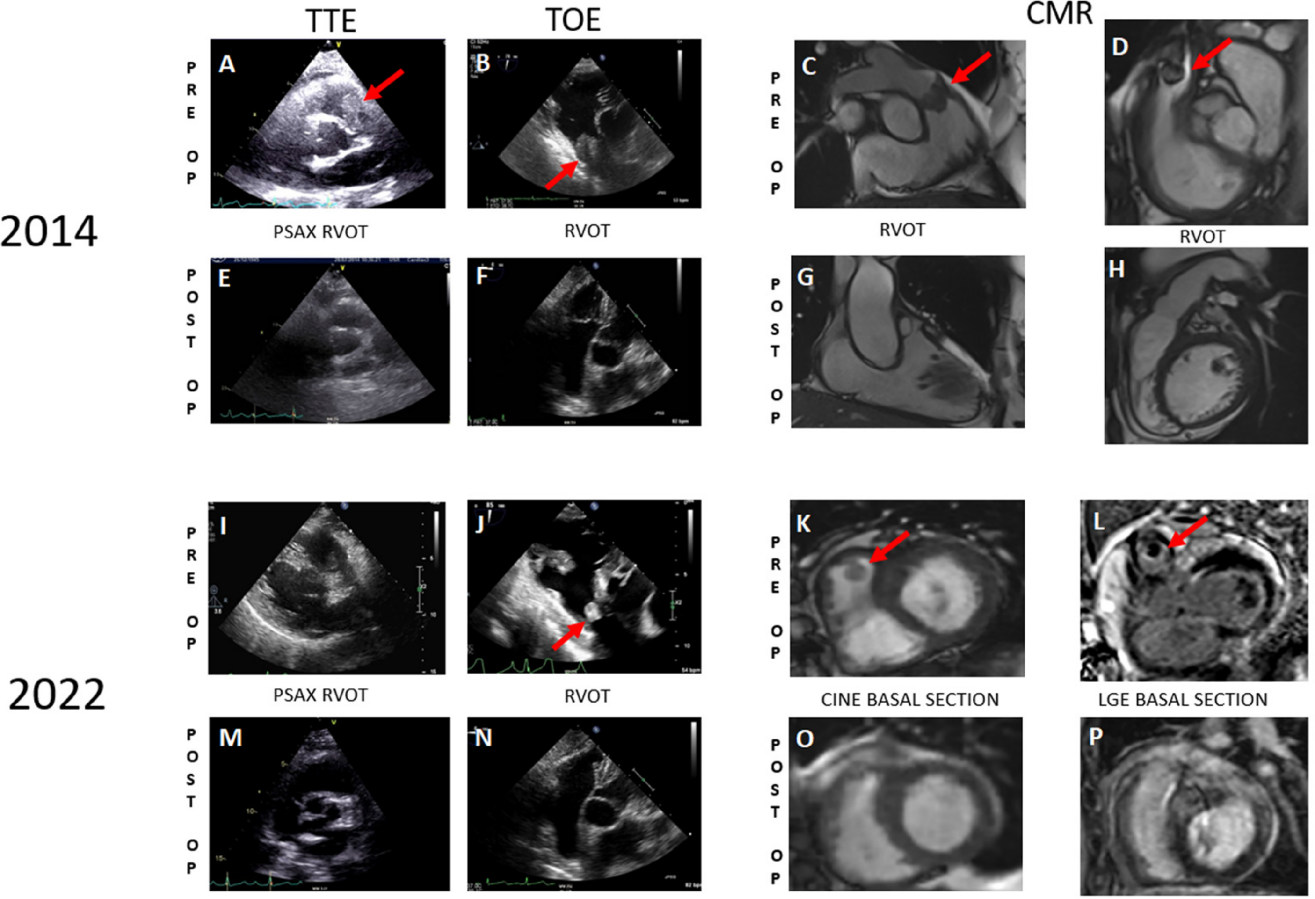
Preoperative cardiac imaging from 2014 to 2022 depicts the initial occurrence of pulmonary artery sarcoma and its recurrence, respectively (**A–D, I–L**) Postoperative imaging from 2014 to 2022 reveals the absence of any lesions (**E–H, M–P**).

**Figure 2:**
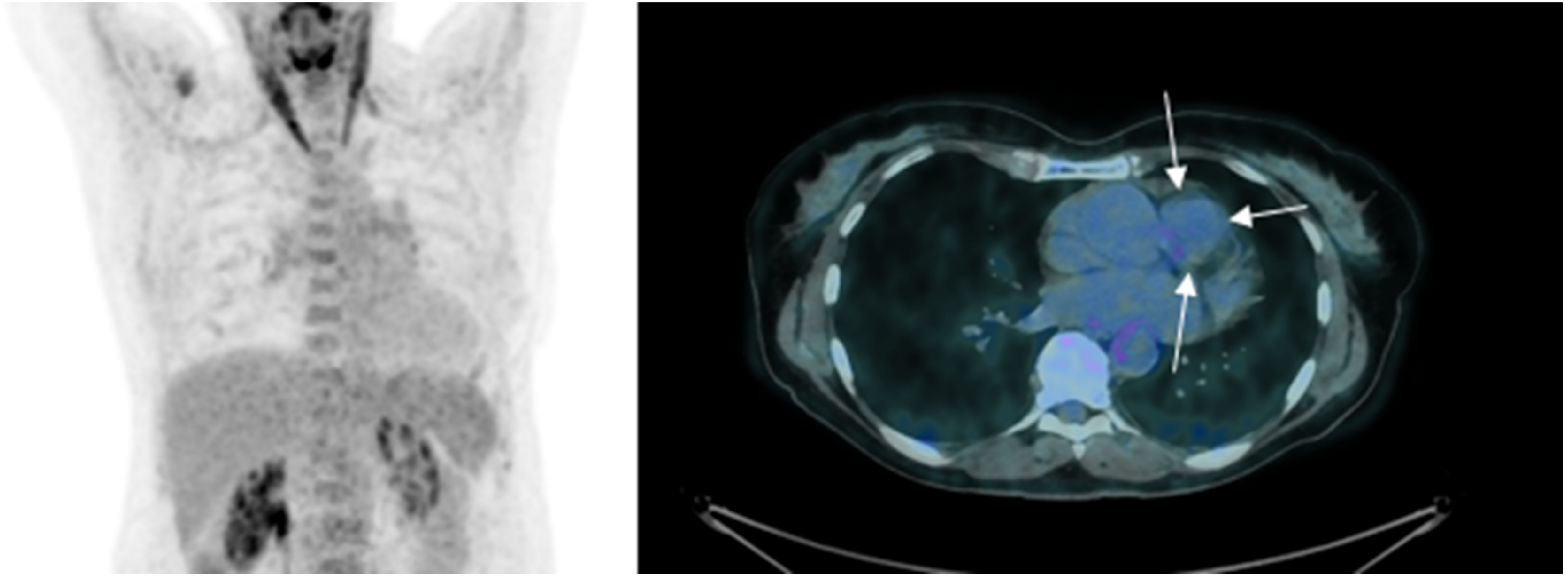
18-FDG PET/CT 2014—initial PET imaging reveals a low metabolic activity of the tumor (indicated by white arrows), comparable to that of the mediastinal blood pool [with a maximum standardized uptake value (SUVmax) of 2.6 for the tumor vs. 2.4 for the blood pool]. Please note that myocardial glucose metabolism is suppressed due to a diet low in carbohydrates and high in fats in the days leading up to the examination.

The patient initially presented in 2014, reporting progressive fatigue and exertional dyspnea classified as NYHA II over a 6-month period. During a cardiac examination, a grade 3/6 crescendo-decrescendo systolic murmur was identified. Transthoracic echocardiography (TTE) revealed moderate pulmonary valve stenosis due to a protruding mass in the right ventricular outflow tract (RVOT), further confirmed by transesophageal echocardiography (TOE) (Figures 1A,B). Cardiac computed tomography (CT) and magnetic resonance imaging (MRI) verified a 25 × 35 × 24 mm mass attached to the RVOT, extending into the pulmonary trunk through the pulmonary valve without apparent invasion of the bifurcation (Figures 1C,D). To characterize the nature of the right atrial mass, a whole-body 18-fluorodéoxyglucose positron emission tomography computed tomography (18FDG PET/CT), performed after 24 h of dietary preparation and heparin pre-administration to suppress physiological myocardial FDG uptake (1), revealed low metabolic activity of the tumor (white arrows), comparable to the mediastinal blood pool [maximum standardized uptake value (SUVmax) of the tumor 2.6 vs. 2.4 for the blood pool] (Figure 2), with no signs of metastasis.

The cardiac mass was partially removed through a longitudinal incision in the pulmonary artery. The mass was very large and adherent to the pulmonary infundibulum but also to the right ventricle up to its apex. Only R2 resection was possible under these anatomical conditions. After resection, there was virtually no pulmonary valve or pulmonary artery wall, which means that the continuity of the right outflow tract has to be rebuild using a homograft. Immediate postoperative TOE demonstrated a proper function of the homograft and no complication, particularly on the tricuspid valve.

**Figure 3:**
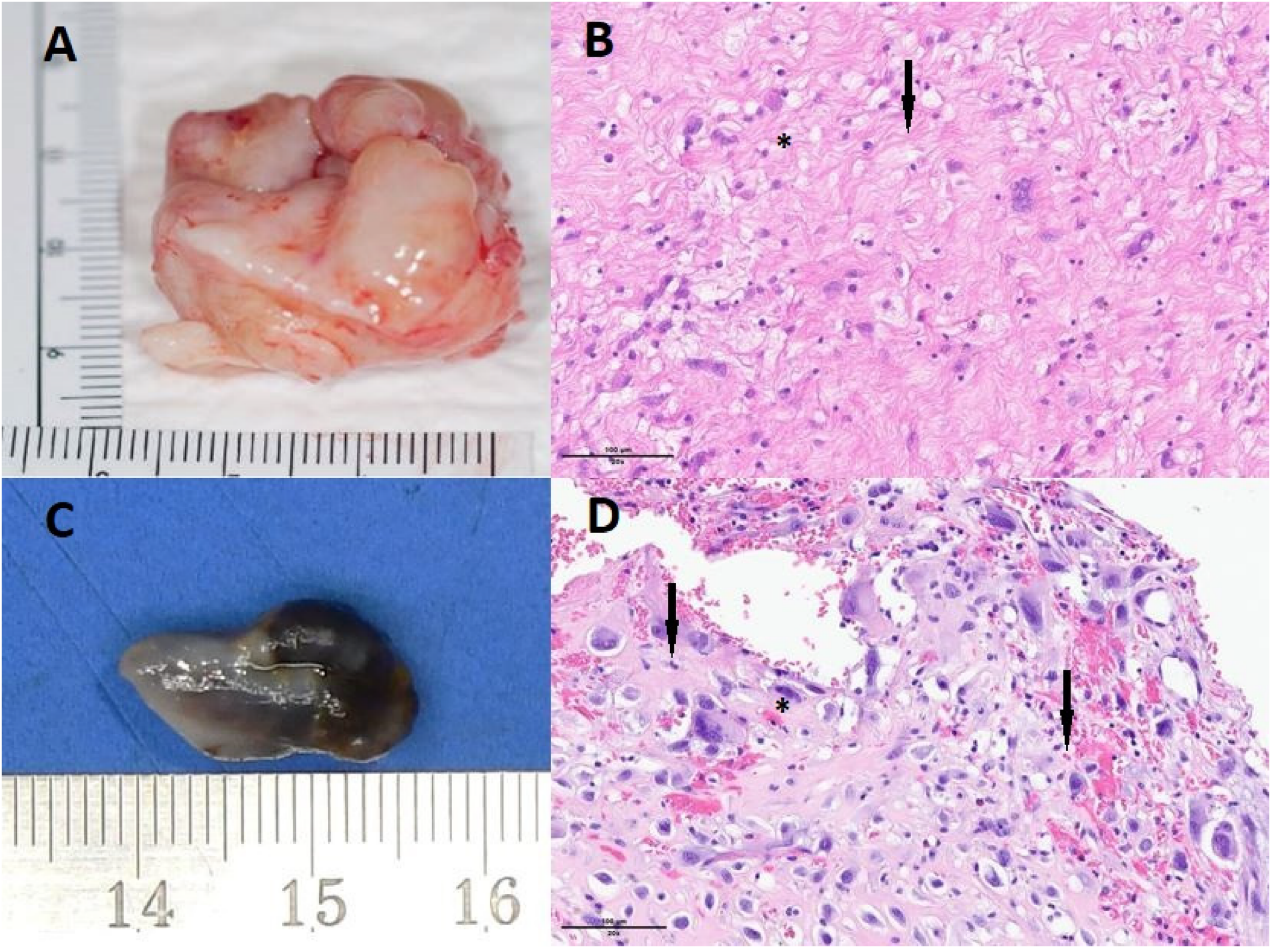
Tumoral lesion resected in 2014 (**A**) displaying scattered high-grade giant cells (**B**, indicated by arrows) within an eosinophilic fibromyxoid stroma (**B**, marked with a star), stained with hematoxylin and eosin (HE) at 200× magnification. Immunohistochemicaly, the cells were lightly and focally positive for CD34, and negative for MDM2, HMB45, Melan A, S100, pancytokeratin, EMA, desmine, SMA, caldesmon and CD99. MDM2 was not amplified in FISH. Tumoral lesion resected in 2022 (**C**), measuring approximately 1.5 cm, exhibits a nodular appearance with a smooth surface, appearing brownish with hemorrhagic foci but lacking obvious necrosis. The tumor is well-encapsulated by a fibrous capsule. Anatomopathology with HE staining revealed tumoral cells displaying numerous atypia and mitosis, along with scattered high-grade giant cells (**D**, indicated by arrows) within an eosinophilic stroma (**D**, marked with a star). Some of these cells were multinucleated (**D**, indicated by arrows). Immunohistochemistry did not demonstrate a specific line of differentiation, with a negativity of the following markers: CD31, CD34, ERG, MDM2, Calrétinine, MYC, CD68 et CD163. HE staining at 200× magnification.

Anatomopathological analysis indicated a proliferation of poorly differentiated malignant tumor cells within a fibromyxoid stroma (Figures 3A,B). The proliferation included giant cells with clarified, vacuolated, or eosinophilic cytoplasm. The nuclei were large, often multinucleate, with marked pleomorphism. Prominent nucleoli were observed in some cells, along with intranuclear inclusions. Immunohistochemicaly, the cells were positive for CD34 and negative for HMB45, Melan A, S100, pancytokeratin, EMA, desmine, SMA, caldesmon and CD99. MDM2 was not amplified in FISH. A diagnosis of undifferentiated pleomorphic cardiac sarcoma was established.

Following radio-chemotherapy with cisplatin (Radiotherapy of 45 Gy—25 fractions of 1.8 Gy—followed by a boost of 12 Gy—6 fractions of 2 Gy—and cisplatin 40 mg/m2, 1×/week for 5 weeks), the patient experienced long-term remission with a good quality of life. Cisplatin therapy was used as a radio-sensibilizing treatment. Subsequent follow-ups with TTE, TOE, and MRI (Figures 1E–H) showed no signs of recurrence.

In 2022, the patient reported a recurrence of progressive dyspnea classified as NYHA II during a follow-up. Upon admission, vital signs included a blood pressure of 144/62 mmHg (mean pressure, 89 mmHg), a resting heart rate of 64 beats per minute, and a transcutaneous oxygen saturation of 97% while breathing ambient air. A systolic pulmonary murmur (3/6) radiating on the carotid arteries was detected during cardiac auscultation. The patient did not exhibit signs of cardiac failure (Killip I), and the neurological examination was normal.

TTE and TOE revealed a resurgence of pulmonary stenosis with a mobile mass measuring 10 × 12 mm in the RVOT (gradient 45/30 mmHg vs. 20/13 mmHg previously) (Figures 1I,J). Cardiac MRI confirmed a T1 isointense, T2 hyperintense pedunculated mass measuring 11 × 11 mm in the RVOT (Figures 1K,L). A differential diagnosis of thrombus was considered, and anticoagulation was initiated. A follow-up MRI after 2 months indicated stability of the mass. Endovascular biopsy attempts were unsuccessful. Multidisciplinary discussions led to the decision for surgical resection of the mass.

The procedure consisted of a transverse incision of the homograft in order to remove the mass which obstructed the pulmonary valve at each systole. The homograft showed signs of degeneration in the form of mild stenosis and insufficiency, but it was decided not to replace the prosthesis, given the complexity of that surgery and the patient's oncological prognosis.

**Figure 4:**
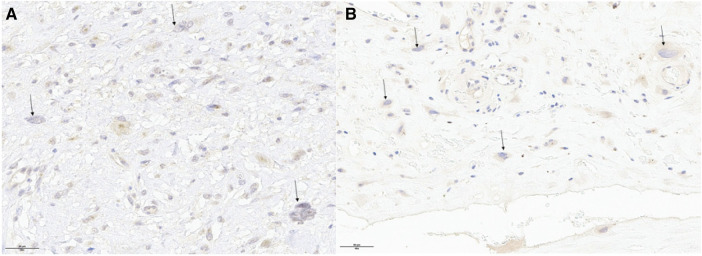
The same morphology and immunohistochemical profile were found in 2014 (panel **A**) and in 2022 (panel **B**) with high-grade giant cells that were negative for MDM2.

Macroscopically, the mass exhibited a nodular aspect with a smooth surface, brownish with hemorrhagic foci but without obvious necrosis (Figure 3C). Anatomopathological analysis revealed a well-circumscribed tumor encapsulated by fibrous tissue. Tumoral cells exhibited numerous atypia and mitosis, with scattered giant high-grade cells (Figure 3D, arrows) within an eosinophilic stroma (Figure 3D,*). Some cells were multinucleated (arrows). Immunohistochemicaly, the cells were negative for CD31, CD34, ERG, MDM2, Calretinin, MYC, CD68 and CD163. MDM2 was not amplified in FISH as in the previous biopsie. The same morphology and immunohistochemical profile were found in 2014 and in 2022 with high-grade giant cells that were negative for MDM2 (Figure 4). In order to search for a clonal link between the two tumor (2014 and 2022), we performed next-generation sequencing on both tissues and we found the same *TP53* exon 5: mutation c.503A>G (p.His168Arg) in both tumors. Given the very similar localization, morphology, immunohistochemical and genomic profile, a diagnosis of recurrent undifferentiated pleomorphic pulmonary artery sarcoma was confirmed.

### Follow-up—patient perspective

After surgical resection, multidisciplinary discussions led to an observational approach, without the implementation of adjuvant treatment. The patient remains asymptomatic, except for NYHA I dyspnea. The cardiac MRI and PET-CT performed at the 3-month follow-up did not reveal signs of new recurrence of the pleomorphic sarcoma (Figures 1M–P).

At the 10-month follow-up, there was evidence of tumoral progression, manifested as a 7 mm thickening of the right pulmonary artery. As the patient's frailty was too important for a third sternotomy, it was decided to perform a pulmonary artery angioplasty to decrease the patient's dyspnea and she received pazopanib treatment (anti-VEGFR). However, this treatment was discontinued due to intolerance a few weeks after initiation. The patient currently declines oncology treatment.

## Discussion

With an incidence ranging from 0.001% to 0.03% in autopsy series, primary cardiac tumors remain exceptionally rare, carrying a poor prognosis (2). Among these, sarcomas represent the most prevalent form of malignant primary cardiac tumors. Notably, sarcomas originating in the pulmonary arteries constitute the rarest subset within primary cardiac sarcomas.

Since its initial description by Mandelstamm in 1923, approximately 300–400 cases of pulmonary arterial sarcoma (PAS) have been reported. Diagnosing PAS remains a challenge as it is frequently misidentified as pulmonary thromboembolism (3). Cardiac sarcomas arise from primitive pluripotent interstitial cells with the capacity for multiple differentiations. The symptoms are nonspecific, resembling those of various other heart diseases, making the diagnosis of cardiac tumors notably challenging.

The diagnosis heavily relies on multimodality imaging, incorporating echocardiography, cardiac computed tomography, and magnetic resonance. However, a definitive diagnosis and treatment hinge on histology, ultimately confirmed through anatomopathology.

The typical onset age for cardiac sarcoma is 45–55 years old, and the female-to-male ratio is 2:1.

The median overall survival for cardiac sarcoma patients is approximately 6 months based on retrospective series (4). Without surgical treatment, the median survival time is a mere 1.5–3 months for pulmonary artery sarcoma (5). However, with “complete” surgical resection, survival improves to 36.5 ± 20.2 months, and with “incomplete” surgical resection, it extends to 11 ± 3 months (6).

Surgical resection remains crucial for the long-term survival of cardiac sarcoma patients, despite the challenges posed by these tumors, such as their location, achieving complete resection, and addressing sarcoma margins. Combining surgical resection with radiotherapy and chemotherapy appears to offer the best prospects for a good quality of life among patients. Recent studies have not demonstrated survival benefits in the last cohorts examined (7). Given the rarity of achieving R0 resection in this type of surgery, active monitoring for disease recurrence is imperative.

## Conclusion

To the best of our knowledge, our case represents one of the longest periods of recurrence-free survival documented for a cardiac sarcoma in the current medical literature. Successful outcomes were achieved through surgical resection combined with state-of-the-art radiotherapy and chemotherapy, essential for optimizing both survival and quality of life. This unexpectedly prolonged recurrence-free survival underscores the efficacy of the comprehensive multimodal treatment approach detailed in existing literature, emphasizing the critical role of a multidisciplinary strategy. Given advancements in surgical techniques, personalized radiotherapy protocols, and chemotherapy planning in the contemporary era, it is imperative to reevaluate survival rates for this rare tumor through new studies.

## Data Availability

The original contributions presented in the study are included in the article/Supplementary Material, further inquiries can be directed to the corresponding author.
